# Structural biology of the Atg8 and Atg12 conjugation systems

**DOI:** 10.1080/27694127.2023.2277582

**Published:** 2023-11-10

**Authors:** Nobuo N. Noda

**Affiliations:** a Institute for Genetic Medicine, Hokkaido University, Sapporo, Japan; b Institute of Microbial Chemistry (BIKAKEN), Shinagawa-ku, Tokyo, Japan

**Keywords:** Atg8, Atg12, structural biology, Atg7, Atg3, Atg16, Atg4, Atg5, Atg10, RavZ

## Abstract

Atg8 and Atg12 are ubiquitin-like proteins, conjugated to phosphatidylethanolamine (PE) and Atg5, respectively, through enzymatic reactions similar to ubiquitylation. The resultant Atg8–PE and Atg12–Atg5 conjugates play crucial roles in autophagy. Structural studies have been extensively performed on all Atg proteins (Atg3, Atg4, Atg5, Atg7, Atg8, Atg10, Atg12, Atg16) involved in these conjugation systems. This review summarizes structural studies and discusses mechanisms of conjugation and deconjugation reactions, as well as autophagic functions of the Atg8 and Atg12 conjugation systems.

## Introduction

Autophagy involves lysosome-dependent degradation of cytoplasmic materials to maintain cellular homeostasis. When autophagy is induced, for example by nutrient starvation, isolation membranes (or phagophores) appear in the cytoplasm, which expand and seal into double-membrane structures, called autophagosomes. Cytoplasmic contents, including proteins, organelles, and even microbes, are sequestered into autophagosomes randomly or selectively. The autophagosome then fuses with the lysosome (vacuole in yeast and plant) and the inner membrane, together with sequestered material, is degraded by lysosomal hydrolases [1].

**Figure 1.**
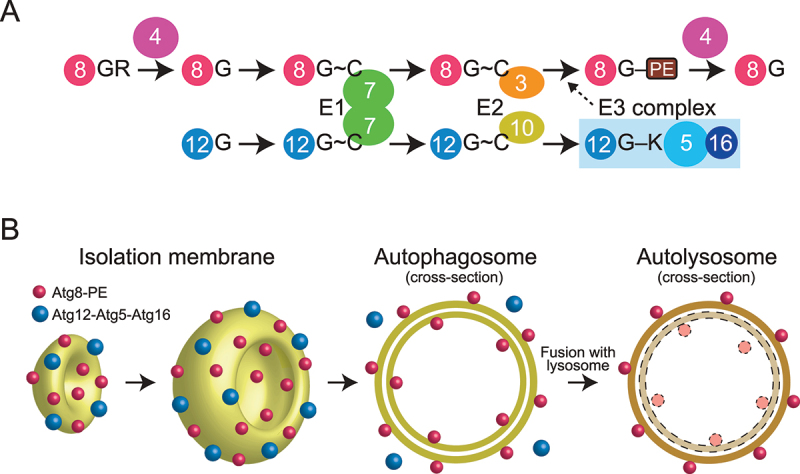
Atg8 and Atg12 conjugation systems. (A) Conjugation reactions of Atg8–PE and Atg12–Atg5 mediated by E1, E2, and E3 enzymes. G, C, and K represent terminal Gly, catalytic Cys, and conjugating Lys, respectively. (B) Localization of Atg8–PE and Atg12–Atg5–Atg16 during autophagosome formation.

Atg8 and Atg12 are ubiquitin-like proteins that were initially identified in budding yeast as essential factors for autophagosome formation (Figure 1) [2,3]. Atg8 is translated as a precursor protein that is processed by the cysteine protease Atg4 to expose Gly116 at the C-terminus. Atg8 Gly116 is adenylated using ATP and then forms a thioester bond with the catalytic Cys of Atg7, which is the E1 enzyme for Atg8 and Atg12. Atg7 transfers Atg8 to the catalytic Cys of Atg3 (the E2 enzyme for Atg8). Finally, Atg3 transfers Atg8 to a phosphatidylethanolamine (PE) to produce the Atg8–PE conjugate with the help of an Atg8-specific E3 enzyme, consisting of Atg12, Atg5, and Atg16 [1,4]. Atg12 is translated with Gly186 exposed at the C-terminus. Atg12 Gly186 forms a thioester bond with the catalytic Cys of Atg7 using ATP. Atg7 transfers Atg12 to the catalytic Cys of Atg10—the E2 enzyme for Atg12. Finally, Atg10 transfers Atg12 to the Lys149 side chain of Atg5 to produce the Atg12–Atg5 conjugate without the help of an E3 enzyme [1,5]. The Atg12–Atg5 conjugate forms a stable complex with Atg16 and functions as the E3 enzyme for Atg8.

Atg8–PE tightly associates with autophagic membranes, which include isolation membranes and inner and outer membranes of autophagosomes, with no preference for membrane curvature. By contrast, Atg12–Atg5–Atg16 preferentially associates with the convex surface of isolation membranes, detaching when isolation membranes mature into autophagosomes [6,7]. Therefore, some populations of Atg8–PE are sequestered into the autophagosome lumen, delivered to lysosomes, and degraded, whereas Atg12–Atg5–Atg16 is not delivered to lysosomes and believed to recycle for autophagosome formation (Figure 1).

This review summarizes studies on the structure and function of Atg8 and Atg12 conjugation systems, and discusses the mechanism of conjugation and deconjugation, and evaluates the physiological roles of conjugation systems in autophagy. Unless noted, *Saccharomyces cerevisiae* nomenclature is used for autophagy-related genes/proteins throughout the manuscript.

## Atg8 and Atg12, the ubiquitin-like proteins essential for autophagy

**Figure 2.**
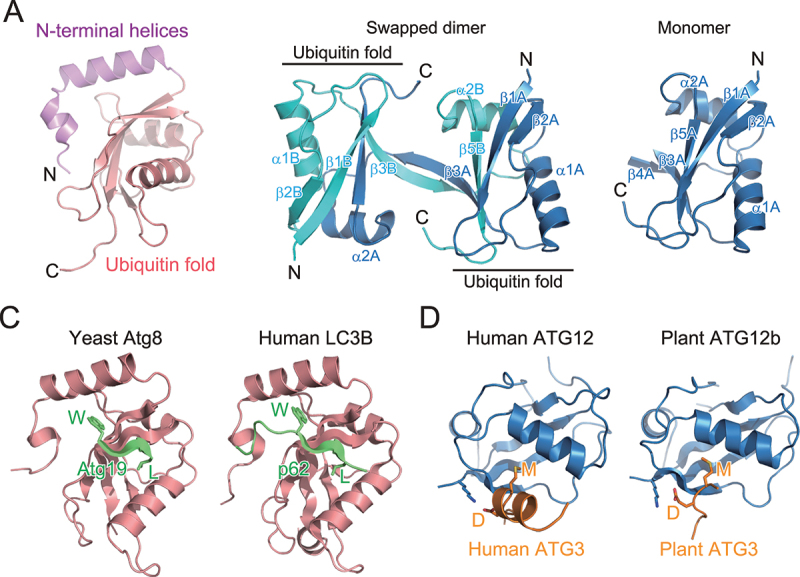
Structure and target recognition of Atg8 and Atg12. (A) Crystal structure of GABARAPL2 (PDB 1EO6). (B) Crystal structure of *Arabidopsis thaliana* ATG12b (PDB 1WZ3). Left, swapped dimer structure. Right, monomer structure reconstructed from the swapped dimer structure. (C) Target recognition by Atg8-family proteins. Left, crystal structure of *S. cerevisiae* Atg8 complexed with a fragment from Atg19 (PDB 2ZPN). Right, crystal structure of *Homo sapiens* LC3B complexed with a fragment from p62 (PDB 2ZJD). (D) Target recognition by Atg12-family proteins. Left, crystal structure of *H. sapiens* ATG12 complexed with a fragment from *H. sapiens* ATG3 (PDB 4NAW). Right, crystal structure of *A. thaliana* ATG12b complexed with a fragment from *A. thaliana* ATG3 (PDB 7EU4).

Atg8-family proteins were the first target for structural studies on the Atg8 and Atg12 conjugation system, because they are small and soluble, and thus, suitable for crystallographic and NMR analyses. The first structure was determined for a mammalian ortholog of Atg8 by X-ray crystallography—GABARAPL2/GATE-16. The structure contained one ubiquitin fold and two N-terminal α-helices (Figure 2A) [8]. Consistently, crystallographic and NMR studies of mammalian ATG8 orthologs and budding yeast Atg8 showed that this structure is conserved across Atg8-family proteins [9-14]. Atg8-family proteins function on the membrane in a PE-conjugated form. NMR studies have shown that GABARAP—chemically-linked to PE in lipid nanodiscs—moves freely on the membrane [15]. Conversely, NMR analysis of Atg8, enzymatically conjugated with PE in lipid nanodiscs, revealed that Atg8 has a preferred orientation on the membrane and shallowly inserts the side chains of Phe77 and Phe79 into the membrane, to cause membrane perturbation *in vitro*, which was proposed to be important for autophagosome formation [16].

In contrast to the great success of structural studies on Atg8, those on Atg12 have been challenging. Ubiquitin and ubiquitin-like proteins are suitable for structural studies due to their small size, stability, and solubility, but Atg12 is unstable and difficult to purify. The structure of plant ATG12b was the first and only determination of Atg12 in a nonconjugated form. However, its structure was determined as a swapped homodimer: the N-terminal portion of ATG12b molecule A (α1A and β1A-β4A) folds into a ubiquitin fold together the C-terminal portion (α2B and β5B) of ATG12b molecule B whereas the C-terminal portion of molecule A (α2A and β5A) folds into another ubiquitin fold together with the N-terminal portion of molecule B (α1B and β1B-β4B), resulting in two ubiquitin folds linked to each other (Figure 2B, left) [17]. This indicates that the folding of Atg12-family proteins in free-form is intrinsically unstable. The monomer structure of ATG12b reconstructed from the swapped dimer structure consists of only a ubiquitin fold (Figure 2B, right).

## Interacting motifs for Atg8 and Atg12

Ubiquitin and ubiquitin-like proteins recognize a conserved sequence (motif) that is widely used by target proteins. Although ubiquitin utilizes a hydrophobic patch, consisting of Leu8, Ile44, and Val70, for interaction with ubiquitin-interacting motifs (UIMs) or domains, Atg8-family proteins do not use this hydrophobic patch for canonical target recognition. Structural studies on yeast Atg8 complexed with a fragment of Atg19 [14] and mammalian LC3B complexed with a fragment of p62/SQSTM1 [14,18] revealed that Atg19 and p62 use the conserved Trp–X–X–Leu sequence to interact with Atg8 and LC3B, respectively (Figure 2C). Notably, Atg8–Atg19 and LC3B–p62 interact in a similar manner: Atg19 and p62 fragments assume an extended β-conformation and form an intermolecular β-sheet with Atg8/LC3B β2. The side chains of Trp and Leu in the Trp–X–X–Leu sequence are bound to hydrophobic pockets formed between α2 and β2 (called W-site or hydrophobic pocket 1) and β2 and α3 (named L-site or hydrophobic pocket 2), respectively. Atg19 and p62 are selective autophagy receptors (SARs) that mediate selective autophagy of vacuolar enzymes and ubiquitinated proteins, respectively [19,20]. Many SARs have been shown to interact with Atg8-family proteins using the consensus core sequence (W/Y/F)–X–X–(L/I/V), where X is any amino acid. This type of sequence is named Atg8-family interacting motif (AIM) or LC3-interacting region (LIR) [21,22]. Because the concave surface of the isolation membrane is decorated with Atg8–PE, SARs promote selective sequestration of their targets into autophagosomes by linking the targets to Atg8–PE at the isolation membrane. Recently, some UIMs were reported to bind to the hydrophobic patch of Atg8-family proteins, which corresponds to the UIM binding site of ubiquitin and is located on the opposite side of the AIM/LIR binding site [23].

In contrast to the diverse binding partners of Atg8, little is known of Atg12-binding proteins. Besides the conjugation target Atg5, Atg3 is the only known binding partner of Atg12. Crystal structures of human ATG12–ATG3 and plant ATG12b–ATG3 complexes [24,25] show an evolutionarily conserved interaction. Human and plant ATG3 conserve the Asp–Met sequence in the ATG12-binding region. Asp–Met binds the hydrophobic pocket between β2 and α1 of ATG12 using the Met side chain and forms ionic interactions with basic residues, such as Lys11 of plant ATG12b and Lys54 of human ATG12, through the Asp side chain (Figure 2D). This short sequence is named Atg12-interacting motif (AIM12) [25]; whether functional AIM12 is conserved in proteins other than Atg3-family proteins is unknown.

## Atg4 and RavZ are deconjugases for Atg8

**Figure 3.**
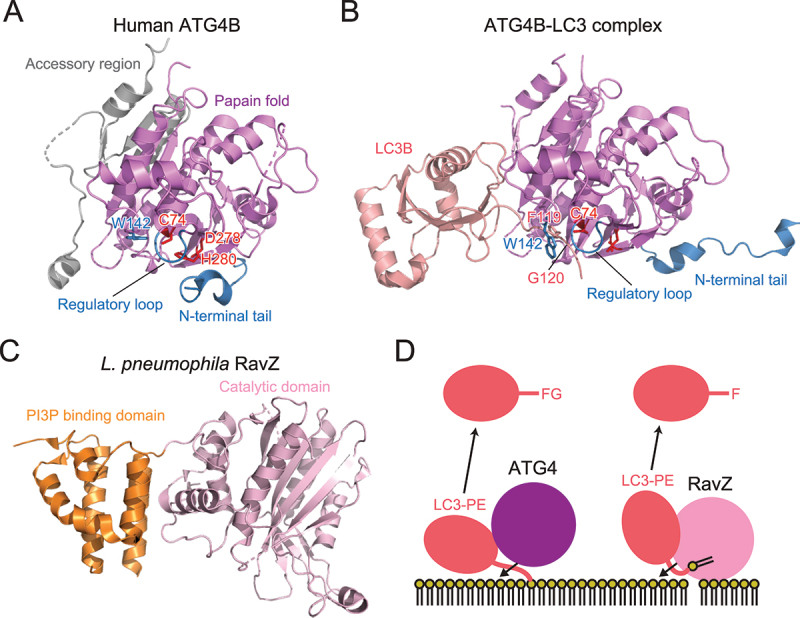
Structure and cleavage mechanism of ATG4 and RavZ. (A) Crystal structure of *H. sapiens* ATG4B (PDB 2CY7). The side chains of the catalytic residues and Trp142 are shown with a stick model. (B) Crystal structure of the *H. sapiens* ATG4B–LC3 complex (PDB 2Z0E). (C) Crystal structure of *L. pneumophila* RavZ (PDB 5CQC). (D) Distinct deconjugation mechanisms of ATG4 and RavZ. FG and F indicate C-terminal residues of LC3 after deconjugation.

The cysteine protease Atg4 is responsible for processing Atg8 precursors and deconjugating Atg8–PE [1]. Because processing of Atg8 precursors is a prerequisite for Atg8–PE formation, Atg4 is essential for both conjugation and deconjugation reactions. Crystallographic analyses revealed that human ATG4B is composed of a papain-like cysteine protease fold and accessory regions [26,27] (Figure 3A). The active site contains the Cys74–His280–Asp278 catalytic triad, which is autoinhibited by the regulatory loop, and the Trp142 side chain. These interact to cover the catalytic Cys74 residue. The N-terminal tail masks the flat surface of ATG4B around the exit of the active site. Upon complex formation with LC3, the regulatory loop is lifted up by Phe119 of LC3 and Gly120 of LC3 binds in proximity to the catalytic Cys74 of ATG4B [28] (Figure 3B). The N-terminal tail of ATG4B undergoes a large conformational change to expose the flat surface around the exit of the active site. These conformational changes allow ATG4B to access membranes and deconjugate LC3–PE. Atg8-family proteins contain a conserved aromatic residue corresponding to Phe119 of LC3, which releases the autoinhibitory conformation of Atg4-family proteins and thereby enables the access of Atg8-family proteins to the active site. ATG4B possesses a LIR motif at the C-terminal tail, which was shown to be important for binding and efficient cleavage of ATG8s [29].

RavZ is a deconjugase for Atg8-family proteins, first identified in *Legionella pneumophila* [30]. In contrast to Atg4-family proteins, RavZ is specific to the lipidated form of Atg8-family proteins and shows little activity toward the precursor form. Moreover, RavZ cleaves the peptide bond at the N-terminal of C-terminal glycine of Atg8-family proteins. Therefore, deconjugation by RavZ irreversibly inactivates Atg8-family proteins. Using this activity, *L. pneumophila* inhibits autophagy in host cells and escapes autophagic degradation. Structurally, RavZ is composed of a catalytic domain, similar to the ubiquitin-like protease family that deconjugates ubiquitin-like proteins, and a phosphatidylinositol 3-phosphate (PI3P) binding domain (Figure 3C) [31]. Furthermore, RavZ possesses intrinsically disordered regions (IDRs) at both termini, which contain three LIRs that mediate interactions with mammalian ATG8 [32]. Using these structural features, RavZ targets to the autophagic membranes through interaction with both PI3P and ATG8s. This activity of RavZ was utilized to study membrane-anchored ATG8 proteins [33,34]. Interestingly, RavZ was proposed to extract the PE moiety of ATG8-PE from the membrane by docking the acyl chains of PE into the lipid-binding site [35]. This is in contrast to Atg4-family proteins, which deconjugate Atg8–PE without extracting the PE moiety from the membrane (Figure 3D).

## Atg7 acts as E1 enzyme for Atg8 and Atg12

**Figure 4.**
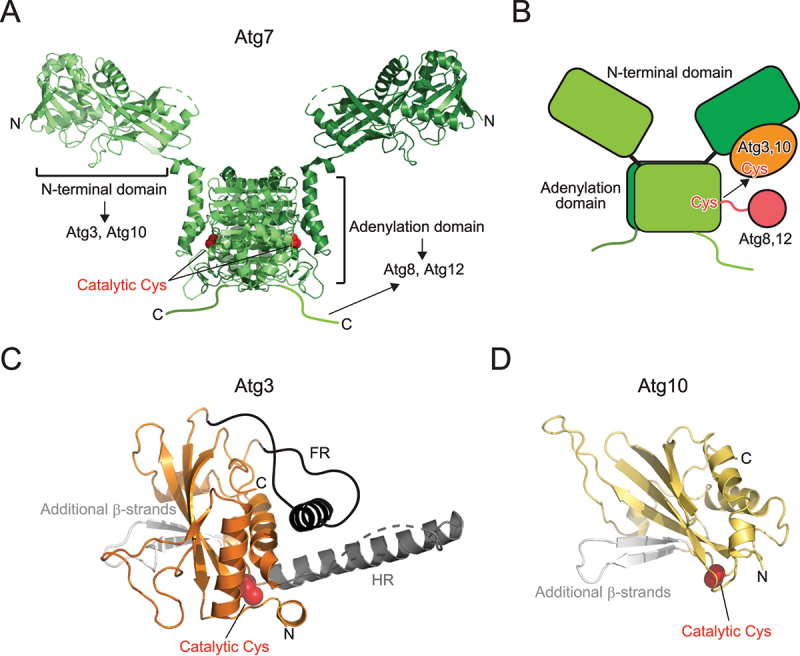
Structure of E1 and E2 enzymes. (A) Crystal structure of Atg7 (PDB 3VH2). (B) *Trans* mechanism of Atg8 and Atg12 transfer from E1 to E2. (C) Crystal structure of Atg3 (PDB 2DYT). Unique insertions FR and HR are colored dark gray and additional β-strands are colored light gray. (D) Crystal structure of Atg10 (PDB 2LPU). Additional β-strands are colored light gray. In (A), (C), and (D), the side chain of the catalytic cysteine is shown with a sphere model.

Although Atg7 belongs to the E1 enzyme family, it is different from canonical E1 enzymes for ubiquitin, SUMO, and NEDD8. Atg7 functions as a homodimer, whereas canonical E1 enzymes function as monomers or heterodimers [36]. Therefore, the functional Atg7 dimer possesses two sets of catalytic Cys, an ATP binding site, and binding sites for E2 (Atg3, Atg10) and ubiquitin-like proteins (Atg8, Atg12). Structurally, Atg7 is composed of the N-terminal domain unique to Atg7 and the C-terminal adenylation domain that is conserved among E1s (Figure 4A) [37-39]. Atg7 forms a homodimer through dimerization of the adenylation domain. Atg7 binds Atg3 and Atg10 using the N-terminal domain and binds Atg8 and Atg12 using the adenylation domain and C-terminal IDR. Structural studies have proposed a multistep recognition model of Atg8 by Atg7: Atg7 first interacts with Atg8 using the C-terminal IDR, transfers Atg8 to the adenylation domain, and finally, accommodates the C-terminal tail of Atg8 at the adenylation site, where thioester formation between the catalytic Cys of Atg7 and C-terminal Gly of Atg8 occurs after adenylation [37,40]. Structural studies on Atg7–Atg3 and Atg7–Atg10 complexes showed that the catalytic Cys of Atg3/Atg10 bound to the N-terminal domain of one Atg7 protomer is in proximity of the catalytic Cys of another Atg7 protomer within a dimer [41,42]. Together with biochemical data, a *trans* mechanism is proposed for the transthioesterification reaction between the catalytic Cys residues of E1 and E2, for which homodimeric architecture of Atg7 is essential (Figure 4B) [37,38,41,42].

## Atg3 and Atg10 act as E2 enzymes for Atg8 and Atg12

Canonical E2 enzymes conserve the E2 fold composed of a four-stranded β-sheet and four α-helices. Both Atg3 and Atg10 have the E2 fold but contain two β-strands instead of two C-terminal α-helices, constituting a six-stranded β-sheet together with the four conserved β-strands of E2 (Figure 4C, D) [5,43]. Atg3 possesses two unique insertions: flexible region (FR) and handle region (HR) [43]. FR consists of ~80 amino acids, most of which were dispersed in the electron density map of the crystal. HR consists of one long α-helix and a flexible loop in the crystal of Atg3 alone, but it was dispersed in the crystal of Atg7–Atg3 complex [42]. These observations suggest that both FR and HR correspond to IDRs. FR is responsible for the interaction with Atg7 and Atg12, whereas HR is responsible for the interaction with Atg8 [43]. Atg3 has an amphipathic α-helix at the N-terminus, which regulates the catalytic activity of Atg3 by targeting Atg3 to membranes with high lipid-packing defects [44-46]. The structure of Atg10 is much simpler— composed of the E2 fold and two additional β-strands, which was confirmed by both X-ray crystallography and NMR [5,47]. Atg10 mediates conjugation between Atg12 and Atg5 without E3 enzymes by directly recognizing Atg5 using the additional β-strands [5].

## Atg12–Atg5–Atg16 is the E3 complex for Atg8

**Figure 5.**
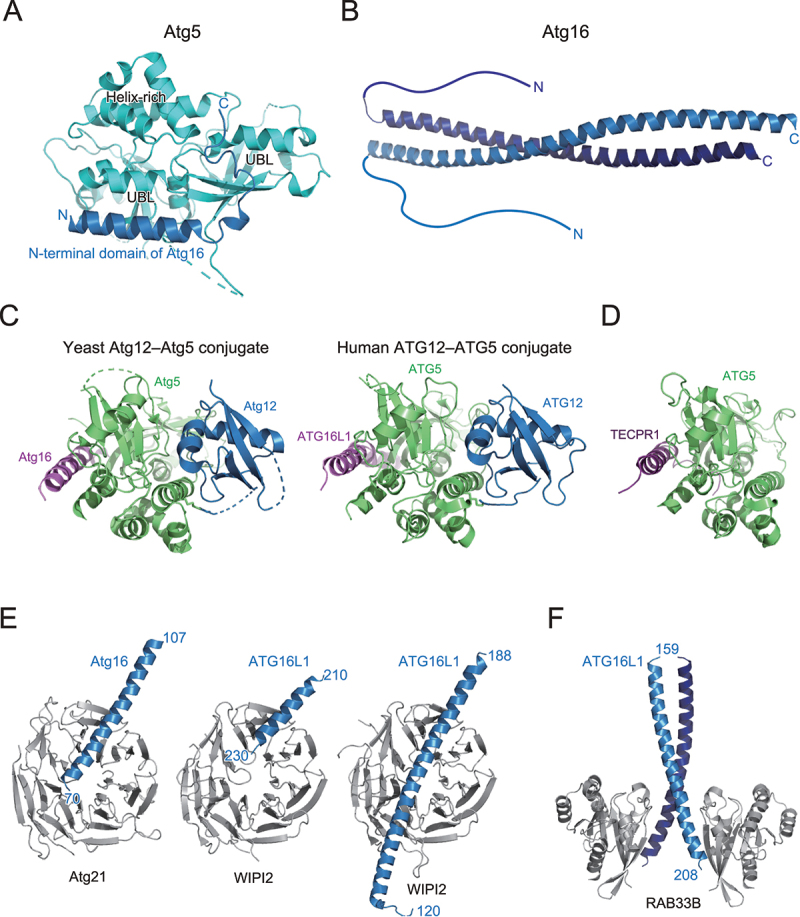
Structure and target recognition of the E3 complex. (A) Crystal structure of Atg5 complexed with the N-terminal domain of Atg16 (PDB 2DYO). (B) Crystal structure of Atg16 (PDB 3A7P). (C) Crystal structure of *S. cerevisiae* Atg12–Atg5 conjugate bound to the N-terminal domain of Atg16 (left, PDB 5CQC) and *homo sapiens* ATG12–ATG5 conjugate bound to the N-terminal domain of ATG16L1 (right, PDB 4NAW). (D) Crystal structure of TECPR1 bound to ATG5 (PDB 4TQ1). (E) Crystal structure of Atg16 bound to Atg21 (left, PDB 6RGO) and ATG16L1 bound to WIPI2 (middle, PDB 7MU2; right, PDB 7XFR). (F) Crystal structure of ATG16L1 bound to RAB33B (PDB 6SUR).

Atg16 is composed of an N-terminal domain and a C-terminal coiled-coil domain, which are important for Atg5 binding and homodimerization, respectively. The crystal structure of Atg5 complexed with the N-terminal domain of Atg16 revealed that Atg5 is composed of two ubiquitin-like domains and one helix-rich domain, which interact to form a globular fold (Figure 5A) [48]. The N-terminal domain of Atg16 folds into an α-helix and binds to the shallow groove formed on the two ubiquitin-like domains. The crystal structure of full-length Atg16 revealed that Atg16 forms a 13-nm long parallel coiled-coil dimer (Figure 5B) [49]. The N-terminal domain is disordered, suggesting that the relative orientation between the N- and C-terminal domains is flexible or that the N-terminal domain is intrinsically disordered when not bound to Atg5. Mutational analyses revealed that the conserved surfaces on the Atg16 coiled-coil dimer are important for autophagy, which later was shown to be the binding region for Atg21 (described below) [49,50]. Crystallographic analyses were successful for budding yeast and human Atg12–Atg5 conjugates using cell reconstitution systems, in which Atg12, Atg5, Atg7, and Atg10 were co-expressed in *E. coli*, which produced sufficient amounts of Atg12–Atg5 conjugates [51,52]. Structures of the budding yeast Atg12–Atg5 conjugate and human ATG12–ATG5 conjugate were determined as a complex with the N-terminal region of Atg16 and ATG16L1, respectively. These two structures are in principle similar to each other: Atg5 tightly interacts with Atg12 using the surface opposite to the Atg16 binding site to form a globular fold, with conserved surfaces in both proteins (Figure 5C). Mutational analyses revealed that the conserved surface is important for E3 activity and autophagy [52]. However, the specific role of the conserved surface in E3 activity is unclear. Recently, some species, such as apicomplexan parasites and *Komagataella phaffii*, were shown to lack Atg10 [53]. In these species, Atg12 and Atg5 form a noncovalent complex and function in autophagy similar to the Atg12–Atg5 conjugate in yeast and human. These observations suggest that conserved surface across Atg12 and Atg5, and not conjugation moiety, is important for autophagy.

In 2012, tectonin β-propeller repeat containing 1 (TECPR1) was identified as a binding protein for the ATG12–ATG5 conjugate in mammals [54]. TECPR1 binds both the ATG12–ATG5 conjugate and PI3P and promotes fusion of autophagosomes with lysosomes. Crystal structure of ATG5 complexed with a fragment from TECPR1 revealed that TECPR1 interacts with ATG5 in a manner similar to the ATG5–ATG16L1 complex, thereby competes with ATG16L1 for ATG5 binding (Figure 5D) [55]. Recently, the ATG12–ATG5–TECPR1 complex was shown to localize and function as E3 for LC3 lipidation at damaged lysosomes, which was suggested to promote autophagosome-lysosome fusion and/or lysosome repair pathway [56].

## Factors that interact with the Atg12–Atg5–Atg16 complex

The Atg12–Atg5–Atg16 complex has at least two roles as the E3 for Atg8 lipidation: one is to interact with Atg3 to activate it, and another is to recruit the Atg8~Atg3 thioester intermediate to autophagic membranes for lipidation. *In vitro* reconstitution and structural analyses have revealed that the interaction of the Atg12–Atg5 conjugate with Atg3 reorganizes the catalytic site of Atg3 to enhance the transfer of Atg8 to PE [57,58]. Although Atg5 and Atg16 could interact with membranes *in vitro* [59], the targeting of the Atg12–Atg5–Atg16 complex to autophagic membranes in cells is mediated by β-propellers that bind polyphosphoinositides (PROPPINs), Atg21 in yeast and WIPI2 in mammals [60,61]. Phosphatidylinositol 3-phosphates, abundant in autophagic membranes, recruit Atg21 and WIPI2, which then recruit Atg12–Atg5–Atg16 and ATG12–ATG5–ATG16L1 complexes, respectively, through interaction with Atg16/ATG16L1. Crystal structures have revealed that Atg21 and WIPI2 interact with the coiled-coil region of Atg16/ATG16L1 in a similar manner (Figure 5E) [50,62,63]. Mammalian ATG16L1 was shown to interact with RAB33B using a region different from the WIPI2-binding region (Figure 5F), which was proposed for recruiting the E3 complex to autophagic membranes or recruiting RAB33B-containing vesicles as a lipid source for autophagosome formation [64,65].

## Roles of the Atg8 and Atg12 conjugation systems in autophagosome formation

**Figure 6.**
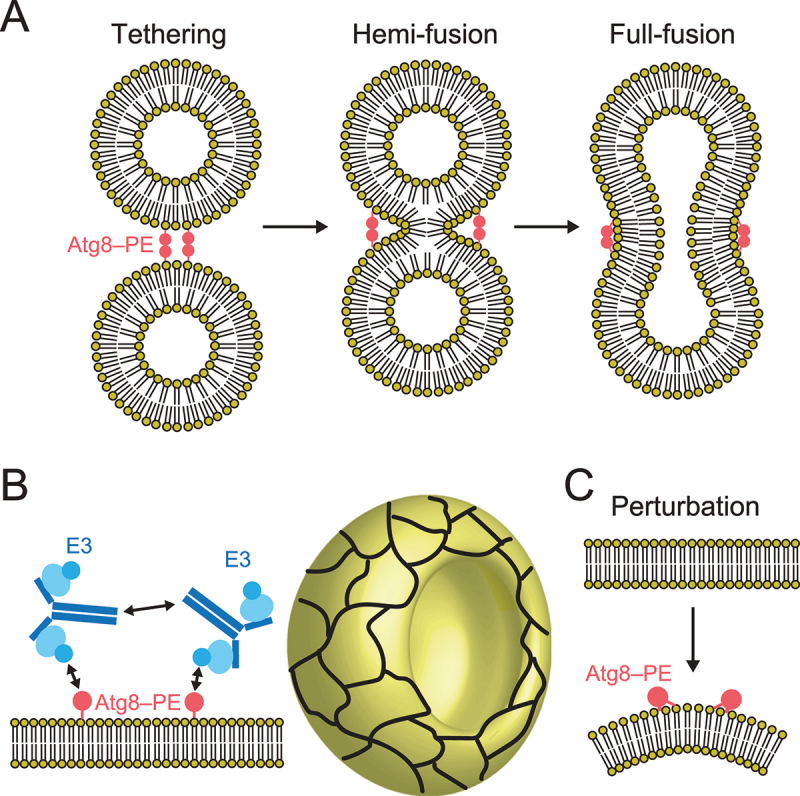
Molecular functions of the Atg8 and Atg12 conjugation systems in membrane dynamics. (A) Membrane tethering, hemi-fusion, and full-fusion activities of Atg8–PE. (B) Proposed model of membrane shaping by Atg8–PE and Atg12–Atg5–Atg16. Cyan ellipse, blue disc, and dark blue lines indicate Atg5, Atg12, and Atg16, respectively. Meshwork on the cup shape (isolation membrane) indicates a higher order assemblage of Atg8–PE and Atg12–Atg5–Atg16. (C) Membrane perturbation activity of Atg8–PE.

The lipidated form of Atg8-family proteins plays crucial roles in cargo recognition during selective autophagy [21,22,66]. What are the molecular roles of the Atg8 and Atg12 conjugation systems in autophagosome formation? Thus far, many researchers have studied the biophysical activities of lipidated Atg8-family proteins *in vitro* and evaluated their membrane tethering, hemi-fusion, and full-fusion activities (Figure 6A) [16,67-70]. Nevertheless, the relationship between these *in vitro* activities and their physiological roles in autophagosome formation is unclear. In 2014, a collaboration model of the Atg8 and Atg12 system was proposed by *in vitro* reconstitution analyses, in which lipidated Atg8 formed a meshwork with the Atg12–Atg5–Atg16 complex on membranes through Atg8–Atg12 and Atg16–Atg16 interactions, which mediate shaping of the autophagosomes (Figure 6B) [71]. In 2021, *in vitro* analyses supported by structural study of Atg8–PE proposed that shallow insertion of aromatic residues of Atg8 into the cytosolic layer induces membrane perturbation, which was shown to be important for efficient autophagosome formation (Figure 6C) [16]. Further studies are required to elucidate specific functions of the Atg8 and Atg12 conjugation systems in autophagosome formation.

## Conclusions

Structural studies on the Atg8 and Atg12 conjugation systems have contributed to establishing the mechanisms of Atg8 lipidation and delipidation, as well as Atg12–Atg5 conjugation. Moreover, the roles of the Atg8 system in cargo recognition during selective autophagy have been established. Considering that as many as eight Atgs, among ~20 core Atgs essential for autophagosome formation, constitute the Atg8 and Atg12 conjugation systems, they must play direct roles in autophagosome formation. Studies must focus on these roles using latest technologies, which include AlphaFold2, to understand the complicated membrane dynamics during autophagosome formation.
